# Factors that associated with TB patient admission rate and TB inpatient service cost: a cross-sectional study in China

**DOI:** 10.1186/s40249-016-0097-x

**Published:** 2016-01-20

**Authors:** Hongyan Hu, Jiaying Chen, Kaori D. Sato, Yang Zhou, Hui Jiang, Pingbo Wu, Hong Wang

**Affiliations:** Center for Health Policy Studies, Nanjing Medical University, Hanzhong Road 140, 210029 Nanjing, P. R. China; Duke Global Health Institute, Duke University, Durham, NC USA; Jiangsu Provincial Center for Disease Control and Prevention, Nanjing, Jiangsu Province China; Zhenjiang Center for Disease Control and Prevention, Zhenjiang, Jiangsu Province China; Hanzhong Center for Disease Control and Prevention, Hanzhong, Shaanxi Province China; Bill & Melinda Gates Foundation, Seattle, WA USA

**Keywords:** Tuberculosis, Admission rate, Service cost, Health care financing, China

## Abstract

**Background:**

China has recently adopted the “TB designated hospital model” to improve the quality of tuberculosis (TB) treatment and patient management. Considering that inpatient service often results in high patient financial burden, and therefore influences patient adherence to treatment, it is critical to better understand the TB patient admission rate and TB inpatient service cost, as well as their influential factors in this new model.

**Methods:**

Quantitative and qualitative studies were conducted in two cities, Hanzhong in Shaanxi Province and Zhenjiang in Jiangsu Province, in China. Quantitative data were obtained from a sample survey of 533 TB patients and TB inpatient records from 2010–2012 in six county designated hospitals. Qualitative information was obtained through interviews with key stakeholders (40 key informant interviews, 14 focus group discussions) and reviews of health policy documents in study areas. Both univariate and multivariate statistical analyses were applied for the quantitative analysis, and the thematic framework approach was applied for the qualitative analysis.

**Results:**

The TB patient admission rates in Zhenjiang and Hanzhong were 54.8 and 55.9 %, respectively. Qualitative analyses revealed that financial incentives, misunderstanding of infectious disease control and failure of health insurance regulations were the key factors associated with the admission rates and medical costs. Quantitative analyses found differences in hospitalization rate existed among patients with different health insurance and patients from different counties. Average medical costs for TB inpatients in Jurong and Zhenba were 7,215 CNY and 4,644 CNY, which was higher than the 5,500 CNY and 3,800 CNY limits set by the New Rural Cooperative Medical System. No differences in medical cost or length of stay were found between patients with and without comorbidities in county-level hospitals.

**Conclusions:**

TB patient admission rates and inpatient service costs were relatively high. Studies of related factors indicated that a package of interventions, including health education programs, reform of health insurance regulations and improvement of TB treatment guidelines, are urgently required to ensure that TB patients receive appropriate care.

**Electronic supplementary material:**

The online version of this article (doi:10.1186/s40249-016-0097-x) contains supplementary material, which is available to authorized users.

## Multilingual abstracts

Please see Additional file 1 for translations of the abstract into the six official working languages of the United Nations.

## Background

Tuberculosis (TB) is a major public health problem in China, with an incidence of 73 per 100,000 and a prevalence of 99 per 100,000 in 2012. More than 890,000 new and relapse cases emerged in 2012, accounting for 12 % of cases globally – a proportion second only to India [1]. In order to reduce financial barriers to treatment, the Chinese government introduced the National TB Control Program (NTP) that incorporated the DOTS strategy and removed fee charges for diagnosis and treatment [2]. Significant improvements have been achieved in the past two decades; the prevalence of smear-positive tuberculosis has fallen by 65 %, bacteriologically positive prevalence declined by 48 %, and prevalence of all pulmonary cases declined by 28 % according to the national tuberculosis prevalence surveys in China [3]. Since 2003, China has adopted a “designated hospital model” in the eastern part of the country to further improve the quality of TB treatment and patient management, under which selected hospitals undertake both TB outpatient/inpatient services and patient management [4, 5]. Recently, policymakers and researchers have being concerned about how the new TB policies will control TB patient admission rates and the cost of TB inpatient services.

In fact, the development of anti-TB drugs has revolutionized treatment in the 1950s, which led to a dramatic reduction in TB case fatality, and since then both developed and developing countries started to abandon hospitalization [6]. Worldwide experiences have demonstrated that it is no longer necessary for TB patients to be isolated from other hospital patients nor from the community with the introduction of short-course chemotherapy and the rapid sterilizing effect of rifampicin in the 1970s [7]. Moreover, the implementation of DOTS has promoted a standard treatment regimen and high completion rates. With a well-functioning DOTS program, higher cure rates and a lower incidence of adverse reactions can be expected [8, 9]. The DOTS program advocates the use of outpatient services in most cases, which can reduce the financial burden of treatment. According to the National TB Control Program Guidelines in China [10], inpatient services are regarded as essential only for severely ill patients, those with comorbidities or associated conditions and patients with adverse reactions.

However, hospitalization of TB patients is common in China. A study using data collected through a national survey of TB specialized hospitals found that the hospitalization rate of TB patients increased 185.3 % from 1999 to 2009, with an associated increase of inpatient service cost. This study estimated that the hospitalization rate in 2010 was 29.8 % in eastern China, 35.6 % in central China and 34.6 % in the west [11]. In 2011, 60.1 % of TB patients covered by the New Rural Cooperative Medical System (NCMS) in Yichang city received inpatient services [12]. In addition, qualitative research from Zhejiang and Guangxi has estimated that nearly 40 % of general TB patients (without comorbidities) experienced hospitalization in the general hospitals [13]. Previous studies have shown no significant differences between inpatient and outpatient treatment outcomes for TB patients, while inpatient costs were substantially higher [14–16]. Tuberculosis inpatients often face high costs, risk of nosocomial infection, loss of income, disruption of family life [17], low therapy completion [18], psychosocial burdens and unnecessary drugs and tests beyond those supplied through the national program [19, 20].

Studies have also found that high treatment costs were associated with higher likelihood of non-adherence [17]. Adherence to TB treatment is crucial for full recovery and to avoid the emergence of drug resistance. Even though DOTS promotes full compliance, high financial burden can result in patients failing to complete the full course of treatment. Due to irregular treatments, smear-negative patients may become smear-positive up to five years later, sometimes developing multiple drug resistant TB (MDR-TB), in turn, infecting others [21].

As hospitalization is often responsible for the high financial burden on patients and for low adherence rates [22], it is important to understand the reasons why health care providers often recommend inpatient service rather than outpatient services for TB treatment. This paper provides estimates of admission rates and medical costs of TB inpatients in two cities in China, then explores the factors that are potentially associated with high admission rates via a mixed method. Finally, recommendations are made as to how policy-makers might act to reduce admission rates and the cost of TB inpatient services, limiting the financial burden on TB patients and hence potentially improving treatment adherence.

## Methods

### Ethical approval

Ethical approval was issued by the Research Ethics Committee of the China Center for Disease Control and Prevention. Informed consent was obtained from all participants prior to the survey and interviews. Data access complied with standard procedure.

### Study design

This study was part of a cross-sectional baseline survey for the China-Gates project Phase II conducted in 2013. We used data obtained in Zhenjiang city of Jiangsu Province in eastern China and Hanzhong city of Shaanxi Province in western China. Counties in each city were grouped into three categories—developed, less developed and least developed according to the gross domestic product (GDP) per capita in 2012 and one county from each category was then randomly selected as the study county. Consequently, Yangzhong (105878 CNY), Dantu (83388 CNY) and Jurong (54140 CNY) were selected in Zhengjiang while Mianxian (24900 CNY), Chengg (22444 CNY) and Zhenba (16383 CNY) were selected in Hanzhong.

### Data collection

#### Patient survey

We carried out a patient survey in the three selected counties to investigate the inpatient service utilization of TB patients. In each county, three townships/streets were randomly selected and, in each, 30 TB cases were randomly selected from the TB case registration list. Patient survey primarily focused on those patients who did not have MDR-TB, started TB treatment in 2012 and completed the treatment or stopped treatment before the survey (April 2013). The content of the questionnaire primarily included socio-economic information of TB patients, pathways of seeking TB care, utilization of services, and medical costs. TB patients were all invited to the township hospital to be interviewed by trained medical students from Nanjing Medical University and Xi’an Jiaotong University. In total, 267 TB patients in Zhenjiang and 270 in Hanzhong completed the questionnaire, and after cleaning cases with missing values, 263 completed questionnaires in Zhenjiang and 270 in Hanzhong were valid.

#### Medical records of TB inpatients from TB designated hospitals

Medical records of TB inpatients between 2010 and 2012, where the primary diagnosis for hospital admission was TB, were collected in six county TB designated hospitals. The main variables of interest in these records were patient age and sex, health insurance coverage, diagnoses at dates of admission and discharge, length of stay, treatment details and related service costs.

#### Qualitative interviews of stakeholders

Key Informant Interviews (KII) and Focus Group Discussions (FGDs) were used to explore the perceptions of stakeholders on the provision and utilization of TB inpatient care. A purposive sampling method was adopted to select the interviewees and FGD participants. All qualitative interviews lasted from fifty to eighty minutes and were both video- and audio- recorded with permission. Semi-structured KIIs were conducted with local health administrators, Center for Disease Control and Prevention (CDC) heads in charge of TB control, CDC TB control unit leaders, heads of TB designated hospitals and local health insurance managers. A total of forty KIIs were completed and all KIIs were conducted in the offices of the interviewees. These KIIs focused on the financial burden on TB patients, patient treatment adherence, health insurance regulation on service provision, perceptions of the provision of TB diagnosis and treatment care, TB diagnosis and treatment practices and payment methods for TB treatment. In addition, fourteen focus groups were formed (eight for TB care providers and six for TB patients), taking place at the designated hospitals. Each TB care provider FGD was comprised of doctors and nurses who treated TB, head of TB units, lab staff and administrative staff associated with TB diagnosis and treatment with 8–10 participants per group on average. The topics for these frontline health care workers covered financial burden on TB patients, patients’ adherence to the treatment, regulation of service provision set by the health insurance agency and its impact on service provision, and doctors’ adherence to the TB practice guideline. The six TB patient FGDs revealed differences in the age and gender of TB patients, treatment experience (inpatient and/or outpatient), financial situation, and classification of TB (new or relapsed cases) with 6–8 participants per group on average. The main points of these discussions for TB patients mainly included: experience with TB treatment; financial burden of TB treatment and its impact on family life; satisfaction with the service provision; perception of TB and MDR-TB infection; reimbursement from health insurance.

#### Document review

We collected the local policy documents relating to health insurance regulations and reimbursement policies from the county level and municipal level health bureaus and health insurance offices.

### Data analysis

Univariate and multivariate analyses were applied to analyze the quantitative data. Firstly, with the patient survey data, we estimated the admission rate which was defined as the proportion of surveyed patients admitted to the hospital. A binary logistic regression was used to explore the association between these six characteristics of TB patients (gender, age, social health insurance, classification of TB cases, household per capital income and sample sites) and the occurrence of hospitalization by computing odds ratios (ORs), which can be adjusted for each of their effects. Secondly, we linked patient surveys with the inpatient medical records by patient name in order to have precise information on the utilization of inpatient service. We successfully matched 271 cases (169 cases from county level designated hospitals and 102 from municipal level hospitals). In the linked database, ANOVA was used to find difference in average length of stay (ALOS) and average medical cost among the counties in the two study cities. Associations between the characteristics of TB patients and the average medical costs were estimated by multiple linear regression analysis, in which the log of the average medical cost was the dependent variable as the data were skewed. Thirdly, with the database of medical records, TB inpatients were categorized as with or without comorbidities as defined by their treatment history. ALOS and average medical cost of inpatient treatment were compared between these two groups. The criterion for significance was set at *P* < 0.05 based on a two-sided test.

For the qualitative data, all tape-recorded qualitative resources were firstly transcribed by the medical students, then read by the interviewers. Secondly, a thematic framework analysis approach was used to systematically analyze the potential reasons for hospitalization and the TB inpatient service cost. The themes were identified by the interviewers based on the transcripts and their topic guides. Inductive codes were then developed and used to cataloged all the transcripts. Thirdly, the Nvivo10 software package, a computer-assisted qualitative analysis program, was employed to manage the qualitative data. For the fairness and accuracy of the study, verbatim quotations were used in the transcripts of different stakeholders and all qualitative data were coded and then categorized carefully. In addition, a team approach was used in coding, sorting and classifying these qualitative data, which provided a form of researchers’ triangulation. Charting was used to identify common and divergent perceptions. Explanations of the emergent patterns in this data were developed.

In addition, we reviewed local policy documents and summarized the policies related to service provision and reimbursement specifically for TB patients, such as regulation of average medical cost for inpatient service, payment method for TB service provision, and reimbursement rates of outpatient and inpatient services.

## Results

### Demographics and health insurance policy for TB treatment

Survey data was obtained for 533 TB patients (263 in Zhenjiang, 270 in Hanzhong). About 57 % of patients from Zhenjiang and 48 % of those from Hanzhong were aged 60 and above, while the proportions of those under 30 were 5.7 and 5.2 % in Zhenjiang and Hanzhong respectively. A large majority were men (73 % in Zhenjiang, 78 % in Hanzhong). Almost 87 % were covered by the NCMS (82 % in Zhenjiang, 92 % in Hanzhong), 8.8 % by the Urban Employee Basic Medical Insurance (UEBMI), and 2.6 % by the Urban Resident Basic Medical Insurance (URBMI). Of the 533 patients, 411 were new patients and 122 were relapsed patients. Almost half of the TB patients in Zhenjiang were from families with higher levels of income. The percentages of TB patients from the low level of household per capita income were 20 % in Zhenjiang and 46 % in Hanzhong (Table 1).

**Table 1:** Demographic and social characteristics of the 533 TB patients fom Zhenjiang and Hanzhong in 2012, (N/%)

Characteristics	Total	Zhenjiang	Hanzhong
Gender			
Male	403/75.6	192/73.0	211/78.1
Female	130/24.4	71/27.0	59/21.9
Age			
15-	29/5.4	15/5.7	14/5.2
30-	67/12.6	24/9.1	43/15.9
45-	157/29.5	73/27.8	84/31.1
60-	280/52.5	151/57.4	129/47.8
Social health insurance^a^			
UEBMI	47/8.8	33/12.5	14/5.2
URBMI	14/2.6	9/3.4	5/1.9
NCMS	463/86.9	215/81.7	248/91.9
Uninsured	9/1.7	6/2.3	3/1.1
Classification of TB cases			
New patients	411/77.1	193/73.4	218/80.7
Relapsed patients	122/22.9	70/26.6	52/19.3
Household per capital income^b^			
Low	176/33.3	51/19.8	125/46.3
Middle	176/33.3	78/30.2	98/36.3
High	176/33.3	129/50.0	47/17.4
Total	533	263	270

Data source: TB patient survey

^a^UEBMI: urban employee basic medical insurance, URBMI: urban resident basic medical insurance, NCMS: new rural cooperative medical system

^b^Household per capital income was classified into three equal parts based on the frequency

Table 2 showed the health insurance policies related to the reimbursement and the payment method of TB treatment from NCMS, in which we found no reimbursement policies or payment methods were drawn specifically for TB treatment in Zhenjaing and Hanzhong. The reimbursement rates for outpatient service ranged from 0 % in Yangzhong to 40 % in Dantu, while 60–90 % for inpatient service in all three counties in Zhenjiang. Patients in Hanzhong can get fixed amounts of reimbursement for outpatient service ranged from 800 CNY in Chenggu to 2500 CNY in Zhenba and the reimbursement rates for inpatient service ranged from 60–80 % in three counties. In addition, inpatients in these six County hospitals should firstly pay the deductible costs and then get the reimbursement with a regulated ceiling cost. Health insurance agency in Jurong regulated the average inpatient service cost so that it would not be higher than 5500 CNY. And Zhenba also specified that average inpatient service cost should not exceed 8,500 CNY in a tertiary hospital and 3,800 CNY in a secondary hospital.

**Table 2:** Description of the health insurance policies related to the reimbursement and payment method for TB treatment from six counties in Zhenjiang and Hanzhong, respectively, in 2012

Policy	Zhenjiang			Hanzhong		
	Dantu	Yangzhong	Jurong	Chenggu	Mianxian	Zhenba
Specific policy	No	No	No	No	No	No
Policy of reimbursement rate and regulations						
Outpatient service	40 %	No	30 %	800 CNY	2,500 CNY	2,000–2,500 CNY
Inpatient service	60–90 %			65–80 %		
Regulation	Deductible: 300–500 CNY			Deductible: 200–300CNY		
	Ceiling: 80,000–200,000 CNY			Ceiling: 130,000CNY		
Policy of payment method and regulations						
Payment method	Global budget			Fee-for-service		
regulations	Jurong:			Zhenba:		
	Average medical cost: 5500 CNY for inpatient service			Average medical cost for inpatient service: 3800 CNY in secondary hospital and 8500 CNY in tertiary hospital		

Data source: health policy documents related to TB prevention and treatment, issues by agencies of New Rural Cooperative Medical System

### Admission rate and the factors that associated with the high admission rate

#### Admission rate

Around 55 % of the surveyed TB patients were hospitalized at least once during treatment in each of the two cities. Some 17 % of patients in Zhenjiang and 27 % in Hanzhong experienced more than one hospitalization, with an average of 1.39 admissions per patient. In Zhenjiang, significant differences in hospitalization rates existed between the three counties (83 % in Dantu, 43 % in Jurong and 39 % in Yangzhong, *P* = 0.000). However, there were no significant differences between the three counties in Hanzhong (63 % in Mianxian, 57 % in Zhenba and 47 % in Chenggu, *P* = 0.120) (Table 3).

**Table 3:** Admission rate and hospitalization times of the 533 TB patients from six counties in Zhenjiang and Hanzhong, respectively, in 2012

Sites	Total	Inpatients	Hospitalization rate (%)^a^	Mean of admissions times	Distribution of hospitalization times (%)			
					1	2	3	4+
Zhenjiang	263	144	54.8	1.26	82.6	11.8	3.5	2.1
Dantu	84	70	83.3	1.24	84.3	10.0	4.3	1.4
Yangzhong	82	32	39.0	1.25	78.1	18.8	3.1	0.0
Jurong	97	42	43.3	1.29	83.3	9.5	2.4	4.8
Hanzhong	270	151	55.9	1.51	73.5	14.6	6.6	5.3
Chenggu	90	43	47.8	1.37	74.4	16.3	7.0	2.3
Mianxian	89	56	62.9	1.48	67.9	21.4	5.4	7.7
Zhenba	91	52	57.1	1.65	78.8	5.8	7.7	7.7
Total	533	295	55.3	1.39	78.0	13.2	5.1	3.7

Data source: TB patient survey

^a^First time hospitalization from the database of TB patient survey

Hospitalization rate Pearson Chi-Square: *P* = 0.000 for three county hospitals in Zhenjiang; *P* = 0.120 for three county hospitals in Hanzhong

#### The potential factors that associated with the high admission rates from the quantitative data

Table 4 shows the results of the logistic regression analysis with the occurrence of hospitalizations as a dependent variable. Tuberculosis patients covered by the NCMS were more likely to have higher hospitalization rates compared to patients covered by UEBMI (*P.E* = 1.644, *OR* = 5.18, *P* = 0.019). In addition, significant differences were found in the hospitalization rates in three counties in Zhenjiang, and TB patients in Dantu were more likely to be hospitalized. More external associated factors should be explored, as few socio-demographic characteristics of TB patients were associated with hospitalization rate.

**Table 4:** Multivariate analysis of the potential factors that influence admission rates of the 533 TB patients from six counties in Zhenjiang and Hanzhong, respectively, in 2012

Dependent variable = occurrence of hospitalization (yes = 1)			
Independent variables	SE	Odds ratio	*P*-value
Gender (compare with female)	0.225	1.09	0.71
Age (compare with 15–29)		1	0.364
Age-30–44	0.466	0.93	0.881
Age-45–59	0.311	0.65	0.164
Age-60+	0.224	0.72	0.139
Household per capital income (compare with low)		1.00	0.865
Middle	0.28	1.00	0.992
High	0.254	0.9	0.664
Patient type (compare with relapse)	0.231	0.87	0.538
Health insurance (compare with UEBMI)		1.00	0.064
URBMI	0.356	1.01	0.972
NCMS	0.703	5.18	0.019
Sites (compare with Dantu)		1.00	0.000
Yangzhong	0.39	3.93	0.000
Jurong	0.36	0.33	0.002
Chenggu	0.311	0.53	0.043
Mianxian	0.308	0.69	0.235
Zhenba	0.316	1.24	0.499

Data source: TB patient survey

#### The potential factors that associated with the high admission rates from the qualitative data

With the analysis of qualitative interviews, the following were potential factors that drove high TB admission rates. First, although all the studied designated TB hospitals were public hospitals, they needed the revenue from TB care to recover their service costs. The inpatient services were more expensive than outpatient services; therefore, the TB designated hospitals were more likely to admit patients for the monetary benefits. This factor was also confirmed by some stakeholders in the interviews with staff in the CDC: *“Provided the profit of hospitals, overtreatment and over examination is obvious in designated hospitals, not to mention the inappropriate hospitalizations”* (administers from CDC in Yangzhong). Certainly, this preference for admitting more TB patients was constrained by the availability of hospital beds. With limited hospital beds, hospital administrators had to prioritize the distribution of beds between specialties for maximized revenue. As one health administrator commented, “*Hospitalization rate in XX hospital is not high and indeed this hospital does not have many TB inpatients. The bed utilization rate is often beyond 100 %. We have no extra beds for TB patients as the revenue from inpatient TB treatment is relatively low compared to other inpatient services*” (Interview results from local health administrator).

Second, many designated hospital managers and TB care providers considered hospitalization to be a control measure for TB infection. Most physicians insisted that hospitalizing TB patients was needed to control infection, especially for those with smear-positive pulmonary TB. *“Even though tuberculosis is an infectious disease, it is not regarded as a category A infectious disease in China. And it is a good way to reduce the spread of TB by getting the patients, especially those that are smear positive, admitted to hospital”* (a physician in Hanzhong).

Third, it was reported that the high admission rates of TB patients were due to patient request. One reason revealed by these interviews was the high reimbursement rate for inpatient services in the health insurance schemes of NCMS and URBMI. *“We can get more reimbursement if we are hospitalized compared to the outpatient service”* (patient in Chenggu). Another reason was the fact that TB patients worried about the spread of TB infection within the family and in the community. *“I worry that my daughter may get infected with tuberculosis as I live together with my daughter and she is going to give birth to a baby……and it is better for me to live separately or just be admitted into the hospital”* (patient in Mianxian)*.* Given the patients’ requests, doctors will admit TB patients in order to avoid conflicts with patients or family members and to avoid the risk of being accused of malpractice if they did not admit the patients. And some health care providers in Zhenjiang expressed that *“In fact, we should acknowledge that most TB patients bear huge psychological pressure, and when they are diagnosed with tuberculosis, they are afraid of telling the truth to their relatives, neighbors and colleagues…. Therefore, patients do not allow us, wearing white coats and masks, to enter their homes or even their neighborhood to complete the follow-up work. So it is reasonable to understand why some patients ask to be hospitalized.”*

In addition, the high admission rates for TB patients can be associated with the reimbursement ceiling policy as well. The TB doctors who we interviewed in the designated hospital said, *“Sometimes we (TB designated hospital) had to pay if the cost (for inpatient services) was beyond the ceiling level. For some serious (TB) patients, we had to discharge them and then have them admitted again later.”* These readmissions could further increase the financial burden on patients because they must pay a deductible for each admission.

### TB inpatient service cost and the factors that associated with the service cost

#### The cost of inpatient TB services

Using the linked database described above, estimates were made for the average medical cost of TB inpatient services (Table 5). The results displayed that the inpatient service cost in Zhenjiang was higher than in Hanzhong. At the county level in Zhenjiang, the average medical cost in Jurong was significantly higher than in Dantu and Yangzhong, 7,215 CNY compared to 5,161 CNY and 4,809 CNY, respectively. In Hanzhong, service cost for TB inpatients in Zhenba was highest, 4,644 CNY compared to 3,678 CNY and 3,732 CNY in Chenggu and Mianxian.

**Table 5:** Average medical costs and average length of stay of the 169 matched TB inpatients from six disignated hospitals in Zhenjiang and Hanzhong, respectively, in 2012

Sites	Number	Medical costs (CNY) ^a^		ALOS (day) ^b^	
		Mean	SD	Mean	SD
Zhenjiang	67	6171.44	3546.66	15.99	8.32
Dantu	27	5160.53	2143.02	14.96	4.44
Yangzhong	6	4808.93	2991.34	11.83	7.55
Jurong	34	7214.67	4229.48	17.53	10.37
Hanzhong	102	3952.07	1732.11	17.39	8.67
Chenggu	24	3678.39	1368.62	22.38	9.29
Mianxian	52	3732.18	1724.02	14.98	7.32
Zhenba	26	4644.47	1913.91	17.62	8.91

Data source: TB patient survey and medical records of the designated hospitals, a total of 169 cases were successfully matched

^a^ANOVA for county hospitals: Zhenjiang *P* = 0.047, significant difference between Dantu vs. Jurong; Hanzhong *P* = 0.060, significant difference between Chenggu vs. Zhenba, Mianxian vs. Zhenba

^b^ANOVA for county hospitals: Zhenjiang *P* = 0.216; Hanzhong *P* = 0.002, significant difference between Chenggu vs. Mianxian, Chenggu vs. Zhenba

#### The factors that associated with the high cost of inpatient TB services from the quantitative data

With the available quantitative data, we examined the relationship between TB inpatient service cost and the comorbidities based on 845 eligible TB patient medical records from designated hospitals in Jurong and Mianxian. The results showed that lung infection (240 patients, 18.8 %), hemoptysis (95 patients, 7.4 %), tuberculosis pleurisy (93 patients, 7.3 %) and COPD (74 patients. 5.8 %) were among the top ten comorbidities. Almost 79 % of inpatients (665) had more than one recorded comorbidity, while some 23 % (198) had over three (Table 6). The analytical results further demonstrated that contrary to expectations, there were no statistically significant differences in the inpatient costs, as well as the ALOS, between the patients with and without comorbidities (Table 7).

**Table 6:** Frequency of comorbidities and the 10 most common comorbidities of TB inpatients in designated hospitals of Jurong and Mianxian from 2010 to 2012

Comorbidities	Number	Percentage (%)
Number of comorbidities		
0	180	21.3
1	282	33.4
2	185	21.9
≥ 3	198	23.4
Diagnosis of comorbidities		
Lung infection	240	18.8
hemoptysis	95	7.4
Tuberculosis pleurisy	93	7.3
COPD	74	5.8
Hypertension	56	4.4
CHD	52	4.1
Diabetes	52	4.1
Heart disease	43	3.4
Pneumonia	38	3.0
Bronchitis	34	2.7

Data source: medical records from the designated hospital

**Table 7:** Average length of stay and average medical costs of TB inpatients with and without comorbidity in designated hospitals of Jurong and Mianxian from 2010 to 2012

Hospitals	Number	Without comorbidity			With comorbidity			P
		Median	Range	Mean	Median	Range	Mean	
Length of stay								
Jurong	285	14	8–25	23.02	17	9–25	21.18	0.423
Mianxian	560	15	10–22	15.74	14	9–21	14.77	0.254
Service cost								
Jurong	285	7037	4,331–12,362	10536	6849	4,486–11,085	9155	0.880
Mianxian	560	3810	2,387–4,702	3825	3893	2,616–5,965	4874	0.073

Data source: medical records of the designated hospitals

Another factor that could have influenced the cost of inpatient services was ALOS (Table 5). While the average cost of inpatient service in Jurong was significantly higher than in Dantu and Yangzhong, there was no significant difference in terms of ALOS (*P* = 0.216). In Hanzhong, the average medical costs among the three counties also varied greatly and there were also significant differences in ALOS (*P* = 0.002); however, Chenggu county had the longest ALOS with the lowest average cost of inpatient services.

We also tried to find the association between the health insurance policies and the inpatient service cost. Average medical costs for TB inpatients in Jurong and Zhenba were 7,215 CNY and 4,644 CNY, which was much higher than the 5,500 CNY and 3,800 CNY limits set by the NCMS.

In addition, we explored the factors related to the high inpatient service cost for TB treatment via multiple linear regression analysis. In this analysis, we used the log of the average medical cost for TB inpatient care as the dependent variable and the variables in Table 1 as independent variables. However, in addition to the classification of TB cases, no differences could be found in the other subgroups of TB inpatients, which indicated the power of the socio-demographic characteristics we explored to explain that the great variation in average medical cost was limited.

#### The factors that associated with the high cost of inpatient TB services from the qualitative data

In both cities, the qualitative studies indicated general agreement across CDC staff and designated hospital health care providers that higher inpatient costs were usually associated with treatment of patients with comorbidities, given the potential need for a more expensive drug regimen which would be self-paid. *“Half of the total cost of outpatient treatment is free to TB patients if they have no complications. And another half of the cost will be reimbursed by the health insurance. TB patients, in fact, just need to pay 25 % of the total cost. But the part paid out-of-pocket by patients will be higher if they have some complications”* (health care providers in Dantu).

## Discussion

This study examined the TB patient admission rate as well as the costs of TB inpatient services in the sample study sites. The results suggest that both TB patient admission rate and inpatient service cost are high. This is clearly not in line with government policy under which TB patients in China receive free outpatient care (free first-line anti-TB drugs, three free sputum smears and two chest X-rays) [23]. The study also explored the possible factors that associated with TB patient admission rate and TB inpatient service cost. The results showed that the financial incentives, misunderstandings of infectious disease control and failure of health insurance regulation are the main potential factors that associated with the high admission rates and high inpatient service costs of TB patients.

In our quantitative study, TB patients covered by the NCMS or those from Dantu were the only factors associated with admission rate in our quantitative analysis. Given that relatively low reimbursement rate for outpatient service in NCMS, it was possible for TB patients to choose inpatient service, which was verified by our qualitative study. In China, NCMS aims to provide health protection over catastrophic illnesses with limited health insurance fund, and therefore, it normally sets relatively high reimbursement rates for inpatient care but low rates for outpatient services [24]. In addition, the NCMS reimbursed outpatient expenses much less than the UEBMI and UEBMI. Difference in admission rate for TB patients in Zhenjiang can be attributed to the difference in health care accessibility. Dantu is located in an urban area of Zhenjiang, where there are many better general hospitals than the county level designated hospitals. It is rational for TB patients in Dantu to seek service from some other municipal hospital, since previous study found that non-designated general hospitals tend to provide inpatient service before the referral [25]. In contrast, it was due to the limited medical resources that resulted in the lowest admission rate for TB patients in Yangzhong, which was also confirmed by the interviews with hospital manager. In fact, there were only four doctors and eight beds available for TB treatment in the Yangzhong designated hospital.

The average medical cost for TB inpatient service was 6171 CNY in Zhenjiang and 3952 CNY in Hanzhong, which were slightly lower than the national average cost, revealed in a cross-sectional study in 2010 [11]. A potential reason was the limited sample size in this study. In addition, significant differences in medical cost existed among three designated hospitals in Zhenjiang. Results of qualitative study attributed the high medical service costs to the treatment of comorbidities and the extensive use of liver protection drugs. With the limited quantitative data, we failed to analyze the differences in the details and structure of the medical costs among the three counties.

Results from our qualitative study confirmed that economic incentives was indeed associated with TB patient admission rates and the inpatient service costs, which was also revealed by many other studies [13, 26]. In China, the profit motive for hospitals and monetary incentives for physicians are closely aligned [27]. With the decrease in government funding for public hospitals in China, insurance and patient payments are the main sources of income for health facilities [28, 29]. Physicians employed by hospitals are rewarded with bonuses and promotions based on the profits they generate by admitting patients, recommending diagnostic tests, and prescribing medications [30]. This undoubtedly encourages physicians to increase earnings by perverse behaviors. Most importantly, as fee-for-service remains standard practice in China, health care providers typically do not face a financial risk from the provisioning of unnecessary services [31]. Consistent with another study, we also found disagreement between the designated hospital staff and other stakeholders, such as heads of local CDCs officials in charge of TB control, over the ‘profit-orientated’ hospitalizations, which can be attributed to the different funding mechanisms between general hospitals and CDCs [13].

Qualitative study also revealed that the high TB patient admission rates was associated with the misunderstanding of infectious disease control. In FGDs with healthcare providers, three physicians from two cities insisted that hospitalization was necessary for the purpose of infection control. In addition, a considerable number of patients in Zhenjiang demanded inpatient services for fear of infecting relatives. Previous studies have concluded that domiciliary treatment is as effective as hospital treatment [32, 33] and inpatient service often resulted in a greater financial burden on patients which may reduce the likelihood of treatment adherence [17]. Considering the possibility of nosocomial infections and the cost-effectiveness of patient management, WHO also recommended that TB patients should be managed as outpatients when possible [34], a position supported by the Chinese government guidelines on TB care described in the introduction. Therefore, health education programs were in need to increase the knowledge of both patients and the population about TB control, which would help limit patents’ unreasonable demand for inpatient care. A previous study had confirmed healthcare providers’ inappropriate behaviors, regarding the irrational use of anti-tuberculosis drugs, overprovisioning of liver protection drugs and frequent lab tests in designated hospitals [20]. And with regard to healthcare providers’ attitude towards hospitalization, further quantitatively study should focus on the extent to which health care providers followed the standard protocols for TB treatment, such as the admission indicators.

Unexpectedly, no differences were found between patients with and without comorbidities regarding the cost of inpatient service utilization, which was not consistent with our qualitative findings that physicians attributed the high inpatient service to the treatment of TB-related comorbidity. The observed distribution of comorbidities was similar to that in a previous study of TB patients [35]. In fact, it is reasonable to conclude these results, as most of the comorbidities of TB patients in our quantitative study were common symptoms, comorbidities or chronic diseases for TB patients and patients did not need inpatient service. Only patients with tuberculosis pleurisy need special treatment, such as pumping fluid by thoracentesis, which would increase financial burden. However, only 7.3 % of the total population gets this disease. Therefore, it seems likely that this reflects unnecessary inpatient treatment of those without comorbidities.

In addition, health insurance policies were also associated with TB patient admission rates and inpatient service costs. Firstly, we found almost 27 % of TB inpatients in Hanzhong experienced more than one hospitalization. This result can be explained by the qualitative findings that designated hospital providers in Hanzhong argued because TB inpatients needed long periods of inpatient care and incurred high medical expenses they had no choice but to use multiple admissions. Secondly, the average medical costs in Jurong and Zhenba had exceeded that costs regulated by NCMS. The potential reason was the fact that the regulations were for all patients in a hospital and could not work well to control some specific diseases. Thirdly, most TB patients asked for inpatient service, which also can be explained by the reimbursement policy of NCMS. Even though TB patients can get free outpatient treatment, they still need to pay for other drugs, such as liver protection drugs. And the rural health insurance scheme did not cover much of the patient outpatient costs, which may drove TB patients for hospitalization [24]. Therefore, the current health insurance policies failed to encourage the designated hospitals and TB patients to provide or seek the appropriate services.

## Conclusions

With quantitative and qualitative study, we found there were several factors impacting on admission rate of TB patients. Health insurance policies may influence hospital admission. Higher reimbursement rate for inpatient service may lead to higher admission rate. Economic incentive was a big motivation for doctors to make admission decision. Misunderstanding of TB infection may be associated with higher admission rate. However, we didn’t find the differences of inpatient service costs between TB patients with or without comorbidities.

### Policy recommendations

There are potential limitations in this study. The quantitative data we collected only allowed us to estimate the admission rates and the costs of inpatient services for TB patients. Most of the results of the potential factors that associated with admission rates and inpatient costs come from qualitative interviews. The contributions of the qualitative analysis were limited. These mixed types of analyses limited our ability to draw any causal conclusions.

Bearing these limitations in mind, the key findings from this study are still relevant and informative to the development and implementation of the current “TB designated hospital model” policy in China. Based on the results, we would like to propose the following policy interventions that could potentially curb the high TB patient admission rates and inpatient service costs, and as a result reduce the financial burden on TB patients and increase adherence rates to the standard TB treatment. First, increase TB health care providers’, TB patients’ and the general population’s knowledge about TB infection control through various health education programs. Educational programs will potentially increase the acceptability of treating TB patients at the community level with outpatient services. Second, optimize the current TB financing policies, for example by revising reimbursement and provider payment policies relevant to TB treatment, which may help encourage the designated hospitals to provide appropriate medical services and to control the medical expenditure. Third, improve the quality of TB services by advancing TB treatment guideline development and its implementation through strong supportive monitoring and supervision interventions.

## Additional file

Additional file 1:
**Multilingual abstracts in the six official working languages of the United Nations.** (PDF 245 kb)
