# Supplementary material for: Factors that associated with TB patient admission rate and TB inpatient service cost: a cross-sectional study in China
Source: Infect Dis Poverty. 2016 Jan 20;5:4. doi: 10.1186/s40249-016-0097-x (PMC4719743; doi:10.1186/s40249-016-0097-x)
Supplement: Additional file 1: — Multilingual abstracts in the six official working languages of the United Nations. (PDF 245 kb) [file 40249_2016_97_MOESM1_ESM.pdf]

Translation of the abstract into the six official working languages of the United Nations

العوامل المرتبطة بمعدل دخول مرضى السل للمستشفيات وتكاليف خدمة نزلاء المستشفى من مرضى السل: دراسة مستعرضة في "الصين"

"هونجيان هو"، "جيايينج تشين"، "كاوري دي. ساتو"، "يانج تشو"، "هوي جيانج"، "بينجبو وو"، "هونج وانج"

الملخص:

الخلفية: تبنت "الصين" مؤخرًا "نموذج المستشفى المخصصة لعلاج السل" لتحسين جودة علاج مرض السل (المعروف اختصارًا بـTB) والتدبير العلاجي للمرضى. إذا أخذنا بعين بالاعتبار أن خدمة نزلاء المستشفى غالبًا ما تسبب عبئًا ماليًا كبيرًا على المريض، مؤثرةً بذلك على التزام المريض بالعلاج، فمن الأهمية بمكان أن نفهم معدل دخول مرضى السل للمستشفيات وتكلفة خدمة نزلاء المستشفى، إلى جانب عواملهما المؤثرة بهذا النموذج الجديد، فهمًا أفضل.

المنهج: أجريت دراسات كمية ونوعية في مدينتين، "هانتشونج" في مقاطعة "شنشي" و"تشنجيانج" في مقاطعة "جيانجسو"، في "الصين". تم الحصول على بيانات كمية من مسح بالعينة لـ533 مريض بالسل ومن سجلات نزلاء المستشفى من مرضى السل من عام 2010 وحتى عام 2012 في ستة مستشفيات مخصصة للأقاليم، بينما تم الحصول على معلومات نوعية بإجراء مقابلات مع أهم المساهمين (40 مقابلة مع أهم المجيبين، 14 مناقشة مع مجموعات بؤرية) ومراجعات لوثائق السياسات الصحية في مجالات الدراسة. كذلك، طُبقت تحليلات احصائية أحادية المتغير ومتعددة المتغيرات بالتحليل الكمي، بينما طُبق نهج الإطار الموضوعي بالتحليل النوعي.

النتائج: بلغت معدلات دخول مرضى السل للمستشفيات في مدينتيّ "تشنجيانج" و"هانتشونج" 54.8 و55.9 بالمائة على التوالي. لقد كشفت التحليلات النوعية أن الدوافع المالية وسوء فهم مكافحة الأمراض المعدية وفشل أنظمة التأمين الصحي كانت العوامل الأساسية المرتبطة بمعدلات دخول المستشفيات وبالتكاليف الطبية. أما التحليلات الكمية، فقد وجدت فروقًا في معدل العلاج بالمستشفيات بين المرضى ذوي أنظمة التأمين الصحي المختلفة والمرضى من الأقاليم المختلفة؛ حيث كان متوسط التكاليف الطبية في "جورونج" و"تشينبا" 7,215 "يوان" صيني و4,644 "يوان" صيني، مما يعتبر أعلى من الحدود التي وضعها النظام الطبي التعاوني الريفي الجديد، والتي تبلغ 5,500 "يوان" صيني و3,800 "يوان" صيني. لم يتم العثور على فروق في التكلفة الطبية أو في مدة الإقامة بين المرضى المصابين بالمراضة المشتركة وغير المصابين بها في المستشفيات على المستوى الإقليمي.

الاستنتاجات: معدلات دخول مرضى السل للمستشفيات وتكاليف خدمة نزلاء المستشفيات كانت مرتفعة نسبيًا. لقد أشارت دراسات العوامل المرتبطة بهذا إلى أن هناك ضرورة ملحة لتطبيق حزمة من التدخلات، تشمل برامج توعية صحية وإصلاح أنظمة التأمين الصحي وتحسين تعليمات علاج مرض السل، لضمان تلقي مرضى السل الرعاية المناسبة.

Translated from English version into Arabic by Heba Kandel, through

TRANSLATORS
WITHOUT BORDERS

关于中国结核病人住院率和住院费用及其相关影响因素的横断面研究

胡红岩，陈家应，Kaori D. Sato，周杨，蒋辉，吴平波，汪宏

摘要：

引言：近年来，中国开始逐步推广结核病定点医院模式以强化对结核病人的治疗和系统管理。在结核病治疗过程中，住院服务往往会给结核病患者造成巨大的经济负担，并进而影响结核病患者的治疗依从性，所以当前很有必要探讨定点医院模式下中国结核病人的住院率和住院费用情况及其相关影响因素。

方法：本研究以陕西省汉中市和江苏省镇江市为样本点，通过定量调查和定性调查相结合的方法获取相关资料。定量资料主要包括合计533位肺结核患者的抽样调查结果以及来自6个县区级结核病定点医院的2010

年到 2012 年所有结核病患者的住院病案首页。定性资料主要来自利益相关者的访谈（40 位关键知情人访谈，14 组焦点小组访谈）和样本地区医保政策文件资料。本研究中定量资料分析方法包括单因素和多因素分析，并运用主题框架分析法分析定性资料。

结果：镇江和汉中的结核病患者住院率依次为 54.8%和 55.9%。定性分析表明经济激励作用，对结核病防治方法的认识误区以及相关医保政策的失效是推高住院率以及住院费用的重要影响因素。除此之外，定量分析结果表明不同医保类型结核病患者以及来自不同区县的患者住院率存在显著差异。句容和镇巴的结核病患者次均费用为 7215 元和 4644 元人民币，远远高于新农合设置的费用限额（句容 5500 元，镇巴 3800 元）。并且在句容和勉县结核病定点医院中，有无并发症对结核病患者的次均住院费用和平均住院日没有影响。

结论：当前，结核病患者住院率和住院费用相对较高。影响因素的研究结果表明当前有必要采取措施来确保结核病患者得到合适的医疗服务，具体措施包括：开展结核病方式相关的健康教育活动，改革医保相关政策规定以及优化结核病治疗指南等。

Translated from English version into Chinese by Hu Hongyan

Facteurs associés au taux d'hospitalisation des patients tuberculeux et au coût des soins aux patients tuberculeux hospitalisés : une étude transversale en Chine

Hongyan Hu, Jiaying Chen, Kaori D. Sato, Yang Zhou, Hui Jiang, Pingbo Wu, Hong Wang

Résumé

Contexte: La Chine a récemment adopté le modèle des « hôpitaux désignés contre la tuberculose » afin d'améliorer le traitement de la tuberculose et la prise en charge des patients. Sachant que les soins en hospitalisation sont souvent très coûteux pour les patients et influent donc sur l'observance du traitement, il est indispensable de mieux comprendre le taux d'hospitalisation des patients tuberculeux et le coût des soins en hospitalisation, ainsi que les facteurs d'influence correspondants dans ce nouveau modèle.

Méthodes: Des études quantitatives et qualitatives ont été menées dans deux villes chinoises : Hanzhong dans la province de Shaanxi et Zhenjiang dans la province de Jiangsu. Des données quantitatives ont été obtenues à partir d'un échantillon de 533 dossiers de patients tuberculeux, hospitalisés ou non, entre 2010 et 2012, dans six hôpitaux de circonscription désignés. Des informations qualitatives ont été recueillies au moyen d'entretiens avec les principales parties prenantes (40 entretiens avec des informateurs clés, 14 discussions en groupe de travail) et de l'examen des documents de politique de santé publique dans les régions étudiées. Des analyses statistiques univariées et multivariées ont été réalisées pour l'analyse quantitative et l'approche par cadre thématique a été appliquée à l'analyse qualitative.

Résultats: Le taux d'hospitalisation des patients tuberculeux était de 54,8 % à Zhenjiang et 55,9 % à Hanzhong. Les analyses quantitatives ont révélé que les incitations financières, une mauvaise compréhension de la lutte contre les maladies infectieuses et l'échec des règlements d'assurance maladie étaient les principaux facteurs associés aux taux d'hospitalisation et aux frais médicaux. Les analyses qualitatives ont relevé des différences dans les taux d'hospitalisation entre les patients couverts par différentes assurances maladie et les patients de différentes circonscriptions. Le coût moyen du traitement des patients tuberculeux hospitalisés à Jurong et Zhenba était de 7215 CNY et 4644 CNY, ce qui dépasse les limites de 5500 CNY et 3800 CNY fixées par le Nouveau Système médical des coopératives rurales. Nous n'avons trouvé aucune différence de coût du traitement ou de durée du séjour entre les patients présentant des comorbidités et ceux qui n'en présentaient pas dans les hôpitaux de circonscription.

Conclusions: Les taux d'hospitalisation des patients tuberculeux et le coût de leur traitement se sont avérés relativement élevés. L'étude des facteurs connexes a indiqué qu'un ensemble d'interventions comprenant des programmes d'éducation à la santé, une réforme des règlements d'assurance maladie et l'amélioration des directives de traitement de la tuberculose est nécessaire d'urgence pour assurer une prise en charge appropriée des patients tuberculeux.

Translated from English version into French by Suzanne Assenat, through

Факторы, влияющие на госпитализированную заболеваемость туберкулезных пациентов и стоимость их обслуживания в стационаре: кросс-секционное исследование в Китае

Хунгя Ху, Цзяин Чэнь, Каори Д. Сато, Янь Чжоу, Хуэй Цзян, Пингбо У, Хун Ван

Реферат

История вопроса: В недавнем времени для повышения эффективности лечения туберкулеза и ведения пациентов в Китае была принята модель специализированных туберкулезных лечебниц. Учитывая то, что обслуживание в стационаре часто приводит к высоким финансовым затратам, и, следовательно, оказывает влияние на соблюдение предписанного режима лечения, чрезвычайно важно хорошее понимание таких показателей, как госпитализированная заболеваемость туберкулезом и стоимость обслуживания пациентов в стационаре, а также влияющих факторов этих показателей в новой модели.

Методы: В двух китайских городах: Ханчжун в провинции Шэньси и Чжэньцзян в провинции Цзянсу - были проведены количественные и качественные исследования. Количественные данные были получены путем выборочного обследования 533 больных туберкулезом и историй болезни госпитализированных пациентов за 2010-2012 г.г. в шести уездных больницах. Качественная информация была получена посредством проведения интервью с ключевыми заинтересованными сторонами (40 интервью с ключевыми лицами, 14 дискуссий в фокус-группах) и изучения документов по политике в области охраны и укрепления здоровья в районах исследований. В количественных исследованиях применялся как одномерный, так и многомерный статистический анализ, а в качественных исследованиях был использован тематический подход.

Результаты: Норма поступления туберкулезных больных в городах Чжэньцзян и Ханчжун составили 54,8% и 55,9% соответственно. Качественный анализ показал, что ключевыми факторами, влияющими на госпитализированную заболеваемость туберкулезом и медицинские расходы, являются финансовые стимулы, непонимание принципов борьбы с инфекционными заболеваниями и несрабатывание системы медицинского страхования. Количественный анализ выявил различия в уровнях госпитализации между пациентами с различными медицинскими страховками и пациентами из разных округов. Средние медицинские затраты на стационарных туберкулезных больных в Джуронг и Чжэнба составили 7215 юаней и 4644 CNY юаней, что превысило предельные значения 5500 юаней и 3800 юаней, установленные новой сельской кооперативной системой здравоохранения. Не было выявлено никаких различий в медицинских затратах или продолжительности пребывания в стационаре между пациентами с сопутствующими

заболеваниями или без них в больницах окружного значения.
Выводы: Госпитализированная заболеваемость туберкулезом и затраты на лечение пациентов в стационаре были относительно высокими. Изучение сопутствующих факторов показывает, что для обеспечения надлежащего лечения туберкулезных больных срочно требуется принятие комплексных мер, включающих санитарное просвещение, реформу системы медицинского страхования и усовершенствование рекомендаций по лечению туберкулеза.

Translated from English version into Russian by Alena Hrybouskaya, through

Factores asociados al índice de hospitalización de pacientes con TB y al costo de los servicios de pacientes hospitalizados con TB : Un estudio transversal en China

Hongyan Hu, Jiaying Chen, Kaori D. Sato, Yang Zhou, Hui Jiang, Pingbo Wu, Hong Wang

Resumen

Antecedentes: Recientemente China adoptó el "modelo de hospital designado para la TB" para mejorar la calidad del tratamiento de la tuberculosis (TB) y el manejo de pacientes. Teniendo en cuenta que el servicio a pacientes hospitalizados a menudo resulta en una importante carga financiera para el paciente y, por lo tanto, influye en el cumplimiento del tratamiento por parte del paciente, resulta fundamental entender mejor el índice de hospitalización de pacientes con TB y el costo de los servicios de pacientes hospitalizados con TB, así como sus factores influyentes en este nuevo modelo.
Métodos: Se llevaron a cabo estudios cuantitativos y cualitativos en dos ciudades, Hanzhong en la provincia de Shaanxi y Zhenjiang en la provincia de Jiangsu, en China. La información cuantitativa se obtuvo de una encuesta realizada a 533 pacientes con TB y registros de pacientes hospitalizados con TB entre 2010 y 2012 en seis condados de los hospitales designados. La información cualitativa se obtuvo mediante entrevistas a las partes interesadas (40 entrevistas a informantes claves, 14 grupos de discusión enfocados) y análisis de los documentos de políticas de salud en las áreas estudiadas. Para el análisis cuantitativo se aplicaron análisis estadísticos univariados y multivariados, y para el análisis cualitativo se utilizó el análisis temático.
Resultados: Los índices de hospitalización de los pacientes con TB en Zhenjiang y Hanzhong fueron 54.8% y 55.9%, respectivamente. Los análisis cualitativos revelaron que los incentivos financieros, la malinterpretación del control de las enfermedades infecciosas y el fracaso de las reglamentaciones de los seguros médicos fueron los factores fundamentales asociados con los índices de hospitalización y los costos médicos. Los análisis cuantitativos encontraron diferencias en el índice de hospitalización entre pacientes con distinto seguro médico y pacientes de distintos condados. Los costos promedio para pacientes hospitalizados con TB en Jurong y Zhenba fueron 7.215 CNY y 4.644 CNY, lo que es mayor que los límites de 5.500 CNY y 3.800 CNY establecidos por el Nuevo Sistema Médico Cooperativo Rural. No se encontraron diferencias en los costos médicos o duración de la estadía entre pacientes con y sin comorbilidad en los hospitales del condado.
Conclusiones: Los índices de hospitalización de pacientes con TB y los costos de los servicios a pacientes hospitalizados fueron relativamente altos. Los estudios de los factores asociados indicaron que se necesita

urgentemente un paquete de intervenciones, que incluya programas de educación para la salud, reforma de las reglamentaciones de los seguros médicos y mejoras en las pautas para el tratamiento de la TB, para asegurarse de que los pacientes con TB reciban el tratamiento adecuado.

Translated from English version into Spanish by Maria Alejandra Aguada, through

TRANSLATORS
WITHOUT BORDERS
